# Respiratory Syncytial Virus Fusion Glycoprotein Expressed in Insect Cells Form Protein Nanoparticles That Induce Protective Immunity in Cotton Rats

**DOI:** 10.1371/journal.pone.0050852

**Published:** 2012-11-30

**Authors:** Gale Smith, Rama Raghunandan, Yingyun Wu, Ye Liu, Michael Massare, Margret Nathan, Bin Zhou, Hanxin Lu, Sarathi Boddapati, Jingning Li, David Flyer, Gregory Glenn

**Affiliations:** 1 Discovery, Novavax Incorporated, Rockville, Maryland, United States of America; 2 Process Development, Novavax Incorporated, Rockville, Maryland, United States of America; 3 Formulation Development, Novavax Incorporated, Rockville, Maryland, United States of America; 4 Analytical Development, Novavax Incorporated, Rockville, Maryland, United States of America; 5 Clinical Development, Novavax Incorporated, Rockville, Maryland, United States of America; University of Iowa, United States of America

## Abstract

Respiratory Syncytial Virus (RSV) is an important viral agent causing severe respiratory tract disease in infants and children as well as in the elderly and immunocompromised individuals. The lack of a safe and effective RSV vaccine represents a major unmet medical need. RSV fusion (F) surface glycoprotein was modified and cloned into a baculovirus vector for efficient expression in Sf9 insect cells. Recombinant RSV F was glycosylated and cleaved into covalently linked F2 and F1 polypeptides that formed homotrimers. RSV F extracted and purified from insect cell membranes assembled into 40 nm protein nanoparticles composed of multiple RSV F oligomers arranged in the form of rosettes. The immunogenicity and protective efficacy of purified RSV F nanoparticles was compared to live and formalin inactivated RSV in cotton rats. Immunized animals induced neutralizing serum antibodies, inhibited virus replication in the lungs, and had no signs of disease enhancement in the respiratory track of challenged animals. RSV F nanoparticles also induced IgG competitive for binding of palivizumab neutralizing monoclonal antibody to RSV F antigenic site II. Antibodies to this epitope are known to protect against RSV when passively administered in high risk infants. Together these data provide a rational for continued development a recombinant RSV F nanoparticle vaccine candidate.

## Introduction

Respiratory syncytial virus (RSV) is the most common cause of acute lower respiratory infection in infants and young children, and a major disease burden in the elderly. Despite the fact that the RSV virus was characterized half a century ago, there is currently no vaccine for RSV and development has been hampered by vaccine-mediated disease enhancement in children administered a formalin inactivated RSV in the 1960s [1], [2]. Challenges in antigen production, purity, stability, and potency of RSV vaccine candidates have also been impediments to development [3]–[5].

The RSV fusion glycoprotein (F) mediates viral entry into cells and cell to cell fusion, is a target of neutralizing antibodies, and highly conserved between RSV A and B strains [6], [7]. RSV F is produced as a precursor (F0) that is cleaved at Arg109 and Arg136 by cellular furin to three fragments, a shorter F2 polypeptide at the N-terminus covalently linked by two disulfides to a longer F1 polypeptide with an 18 amino acid fusion domain at the N-terminus and a hydrophobic membrane spanning region near the C-terminus; the intervening 27 amino acid fragment is released. Neutralizing monoclonal antibodies palivizumab and motavizumab bind to RSV F antigenic site II (Asn258 - Val278) [8] and have been shown to protect against both lower and upper respiratory RSV disease in high risk and term infants [9], [10] The structures of the RSV F epitope polypeptides that bind these neutralizing antibodies are larger than the linear peptide and palivizumab binds with nanomolar and motavizumab picomolar affinity to RSV F [11]–[13]. Modeling predicts that the full extent of the binding of palivizumab and motavizumab requires amino acids from one or two RSV F protomers, respectively. Therefore preserving RSV F tertiary and quaternary structures may be important in the development of an RSV F vaccine to preserve the native conformation of this important neutralizing region.

In this report an oligomeric form of a modified full length RSV F was efficiently produced in Sf9 insect cells using a baculovirus vector. Recombinant RSV F extracted from cellular membranes and purified, assembled into nanoparticles with morphology consistent with F oligomers in a postfusion conformation [11], [14]. Cotton rats were used to investigate the induction of functional immunity, efficacy and potential safety of an RSV F nanoparticle vaccine candidate.

## Materials and Methods

### Ethics Statement

Male cotton rats (*Sigmoidon hispidus*) 5–6 weeks of age were obtained from Ace Animals, Inc. (Boyerton, PA) and maintained at Covance (Denver, PA). All experimental procedures were in accordance with the NRC Guide for the Care and Use of Laboratory Animals, the Animal Welfare Act and the CDC/NIH Biosafety in Microbiological and Medical Laboratories. This cotton rat study number 2009-0925 was approved by Covance Research Products, Institutional Animal Care and Use Committee (IACUC); Covance Research Products, Inc., 465 Swampbridge Road, Denver, PA 17517.

### Cells, Virus, and Cloning

*Spodoptera frugiperda* (Sf9) insect cells (Invitrogen, Grand Island, NY) were maintained in serum free medium as suspension cultures and the recombinant baculoviruses expressing RSV F genes were generated by using Invitrogen Bac-to-Bac baculovirus expression system as described previously [15]. RSV A2 F sequence (Genbank Accession No.U63644) containing 574 amino acids was codon-optimized for insect cells, synthesized, and cloned into pFastBac1 (Invitrogen), downstream of the *Ac*MNPV polyhedrin promoter (GeneArt, Regensburg, Germany). Briefly, baculovirus bacmid DNA was generated by site-specific homologous recombination following transformation of pFastBac1-based transfer plasmid containing RSV F gene into *E. coli* DH10Bac competent cells (Invitrogen), which contained the *Autographa californica* multinuclear polyhedrosis virus (*Ac*MNPV) genome. Recombinant bacmid DNA was extracted from *E.coli* cells and transfected into Sf9 cells using CellFectin reagent (Invitrogen). The transfection supernatants were harvested and recombinant baculovirus (rBV) plaque purified and amplified. The titers of rBV stocks were determined by using BacPak Baculovirus Rapid Titer Kit (Clontech, Mountain View, CA).

Site directed mutations were engineered into wide-type F furin cleavage sites I and II using the QuikChange site-directed mutagenesis kit (Agilent, Santa Clara, CA ) and deletions were introduced into the RSV F1 fusion domain using specific PCR primers. For example, to introduce mutations into wide-type F furin cleavage sites II of RSV F clone #541 a sense 5′-TCACATTGAGCAAAAAGCAGAAGCAACAGTTTCTGGGCTTCCTCCTGG-3′and complementary anti-sense primer 5′-CCAGGAGGAAGCCCAGAAACTGT-TGCTTCTGCTTTTTGCTCAATGTGA-3′ was used. To delete 10 amino acids (Δ137aa–146aa) in the RSV F clone #541 fusion domain, sense 5′-GAGCAAAAAGCGTAAGCGCAGGGCTATTGCGTCGG-3′ and complementary anti-sense 5′-CCGACGCAATAGCCCTGCGCTTACGCTTTTTGCTC-3′ primers were used to construct clone #683.

### Preparation of RSV and FI-RSV Virus

RSV Long strain (ATCC # VR-26) was propagated in HEp-2 cells (ATCC #CCL-23). HEp-2 cells were grown in EMEM medium supplemented with 10% fetal calf serum. Formalin inactivated RSV (FI-RSV) was prepared in HEp-2 cells by modification of the method of Prince et al, [16]. The medium from T175 flasks of 80% confluent HEp-2 cells was decanted and each flask was inoculated with 10 ml of RSV Long strain. After a 2 hr adsorption period at 36°C the flasks were refed with EMEM, 10% fetal bovine serum, 2 mM L-glutamine and incubated at 36°C for approximately 72 hrs until CPE was 80%–100%. The virus was stabilized by mixing a 10% volume of SPG (2 M sucrose, 110 mM potassium phosphate, pH 7.1, 5 mM monosodium glutamate) to each flask. The virus harvests were pooled and clarified by centrifugation at approximately 1000×g for 20 min at 4°C. The clarified virus was filtered through a 0.8 µm filter, formalin treated by the addition of 1/10 volume of a 1∶400 dilution of 37% formaldehyde in sterile water, and inactivated for three days at 36°C with gentle stirring. The formalin treated virus was pelleted at 100,000×g for 60 min in a Surespin 630 rotor at 10°C and suspended in 1/20 volume of HMEM, 10% SPG. The suspended virus was centrifuged at 1000×g for 15 min 4°C to remove insoluble material then incubated with 4 mg/ml aluminum hydroxide (Alhydrogel) overnight at room temperature. The virus-alum mixture was centrifuged at 1000×g for 15 min, suspended in approximately one half volume of HMEM, 10% SPG, and stored in 0.5 ml aliquots at 4°C. The amount of virus bound to the alum was estimated at 44% by measuring total protein before and after alum adsorption which was estimated to be 6.1×10^8^ plaque forming units per milliliter (pfu/ml).

### FACS Analysis

2×10^6^ cells/ml Sf9 cells were infected at MOI = 1 pfu/cell with rBV, harvested 48 hrs post-infection, washed twice by FACS buffer (PBS, 1% fetal bovine serum). A 1∶500 dilution of anti-RSV F sheep serum was added and incubated at room temperature with cells for 30 min followed by staining with fluorescent PE (phycoerythrin) conjugated donkey anti-sheep IgG (Santa Cruz Biotechnology, Santa Cruz, CA) at 4°C for 1 hr. Stained samples were analyzed using a FACS Calibur (BD Biosciences, San Jose, CA) with CellQuest software (BD Biosciences) and data analyzed with Flowjo (Tree star, Ashland, OR) software.

### Purification of Recombinant RSV F

Cultures of Sf9 cells at 2×10^6^ cells/ml were infected with the recombinant baculovirus (BV683) at MOI = 0.5 pfu/cell and incubated at 27°C for 67 hrs. Infected cells were harvested by centrifugation for 15 min at 4000×g and cell pellets washed with PBS. Recombinant F glycoprotein was extracted from cellular membranes with 25 mM Tris-Cl (pH 8.0), 50 mM NaCl, 0.5% Tergitol NP9 and 2 µg/ml Leupeptin at a volume of 10 ml per 1 g of washed cells and insoluble material removed by centrifugation at 7,000×g for 1 hr. The soluble RSV F protein was purified with a combination of anion exchange, affinity and cation exchange chromatography. Briefly, detergent soluble F was passed through a Fractogel EMD TMAE Hicap (M) anion exchange column equilibrated in 25 mM Tris, 50 mM NaCl, 0.02% Tergitol NP9, pH 8.0. Nucleic acid and protein contaminants bound to the column while RSV F and other host contaminants flowed through. Glycosylated RSV F was further purified by binding to Lentil Lectin Sepharose 4B and elution with 25 mM MES, 100 mM NaCl, 500 mM methyl-alpha-D-mannopyranoside, 0.1% PS80, pH 6.8. Finally, RSV F nanoparticles were bound to a Fractogel EMD SO3^−^ in 25 mM sodium phosphate, 0.1 M sodium chloride, 0.01% PS80, pH 6.8 and eluted by the addition of 0.6 M NaCl followed by 0.22 µm sterile filtration.

### Negative Staining Electron Microscopy

Purified RSV F particles were adsorbed by floatation for 2 min on freshly discharged 400-mesh carbon parlodion-coated copper grids (PolySciences, Warrington, PA). The grids were rinsed with buffer (20 mM Tris, pH 7.4, and 120 mM KCl), negatively stained with 1% phosphotungstic acid, and dried by aspiration. The F protein was visualized using a Hitachi H-7600 transmission electron microscope (Hitachi High Technologies America, Schaumburg, IL) operating at 80 kV and digitally captured with a CCD camera at 1 K × 1 K resolution (Advanced Microscopy Techniques Corp., Danvers, MA).

### Reverse Phase and Size Exclusion HPLC Analysis

To perform reversed phase HPLC, 10 µg of purified RSV F protein was loaded onto a PLRP-S 4000A, 2.1×150 mm column (Varian, Palo Alto, CA). Gradient elution was achieved by two mobile phases. Mobile phase A consists of 0.1% trifluoroacetic acid (TFA) in water and mobile phase B consists of 0.1% TFA in acetonitrile with a flow rate of 0.75 ml/min and column temperature of 75°C. Mobile phase gradient was 20% of B at 0.0 ml/min and ramped to 100% of B at 15 min with a linear gradient then kept at 100% of B for 5 min. The signal was collected using diode array detector at 280 nm. Samples were analyzed by 1200 HPLC system from Agilent (Santa Clara, CA).

For size exclusion HPLC, 5 µg of RSV F protein was loaded onto a BEH200, 1.7 µm, 4.6×300 mm size exclusion column (Waters Corporation, Milford, MA) and an isocratic gradient using a mobile phase of 0.1 M HEPES, 0.4 M NaCl, pH 7.0 at a flow rate of 0.25 ml/min for a total run time of 20 min. The signal was collected using a diode array detector at 280 nm.

### Dynamic Light Scattering

Particle size distribution was determined by dynamic light scattering using a Zetasizer Nano (Malvern Instruments Ltd.). RSV F sample was diluted with PBS and particle size distribution determined by measuring the intensity of light scattered by the particles using a detector placed at an angle of 173°. Samples were measured in duplicate and average values were reported as Z.average of diameter of the particle (d.nm). The polydispersity index (pdi) was monitored as a marker of the width or heterogeneity of the particle and as a measure of multiple species. A polydispersity index value below 0.2 was considered acceptable for the sample to be homogenous and monodisperse.

### Immunization and RSV Challenge in Cotton Rats

The immunogenicity and protective efficacy of purified RSV F nanoparticles was evaluated in the cotton rat model. Animals received 2 vaccinations by intramuscular injection in the thigh on day 0, and 21. Animals (n = 8/group) received either 1 µg, 6 µg or 30 µg purified RSV F with or without 120 µg aluminum phosphate adjuvant (Brenntag AG), FI-RSV or control vehicle. The RSV virus control group was inoculated on day 0 with 100 µl live virus (1×10^6^ pfu) intranasally (50 µl/nare). Sera were collected on day 0, 21 and 49 for serological analysis. RSV challenge was performed on day 49 by intranasal administration of 100 µl (50 µl/nare) live RSV Long strain (1×10^6^ pfu). Cotton rats were housed one animal per cage. Blood was collected via the retro-orbital route and serum was obtained by centrifugation of whole blood. Isolated sera were transferred to labeled tubes and stored at −20°C until tested. Animals were observed daily after challenge for signs of disease until day 54 at which time animals were euthanized and lung tissue collected for virus isolation and histopathology.

### RSV F ELISA

RSV F-specific antibodies in cotton rat sera were evaluated in an enzyme linked immunosorbent assay (ELISA). 96-well MaxiSorp microtiter plates (Thermo Scientific, Rochester, NY) were coated with 2 µg/ml of purified RSV F protein overnight at 2–8°C. Plates were washed with phosphate buffered saline containing 0.05% Tween (PBST, Quality Biologicals, Gaithersburg, MD ) and unreacted surface blocked with Blocking buffer (5% milk in PBS, Quality Biologicals) for one hour at room temperature. Plates were washed with PBST and 2-fold serial dilutions (1∶10 to 1∶1,024,000) of cotton rat sera was added to RSV F protein coated plates in duplicate. As a positive control, anti-RSV F sheep polyclonal antibodies were used at a starting dilution of 1∶30. Plates were incubated for two-hours at room temperature, washed with PBST, and bound antibodies detected by the addition of horseradish peroxidase conjugated goat anti-rat IgG (Southern Biotech, Birmingham, Alabama). After a 1 hr incubation, plates were washed with PBST and 3,3′,5,5′-Tetramethylbenzidine substrate (Sigma, St. Louis, MO). The reaction was stopped by the addition of TMB Stop Buffer (Scytek Laboratories, Logan, UT) and plates read at 450 nm in SpectraMax plus plate readers (Molecular Devices, Sunnyvale, CA). Data was analyzed using SoftMax pro software. A 4-parameter fit curve was used to analyze the data and antibody titer was defined as the reciprocal serum dilution that resulted in an OD_450_ of 1.0.

### Palivizumab Competitive ELISA

Competitive binding of the cotton rat sera with biotin labeled palivizumab monoclonal antibody (ASD Specialty Heath Care Inc., Chicago IL) was performed in 96 well plates. Palivizumab was biotinylated with a labeling kit (Pierce, Rockford, IL) as per manufacturer’s instructions. Nunc MaxiSorp microtiter plates were coated with 2 µg/ml purified RSV F and incubated overnight at 2–8°C. Plates were washed with PBST and blocked with Starting Block buffer (Pierce) at room temperature for one hour. After washing, serial 2-fold dilutions (from 1∶20 to 1∶1280) of sera were prepared in duplicate and spiked with 100 ng/ml of biotinylated palivizumab and added to RSV F coated plates. Plates were incubated for two-hours at room temperature, washed with PBST, and incubated with streptavidin-conjugated horseradish peroxidase (e-Bioscience, San Diego, CA). Plates were incubated for an additional hour, washed with PBST and, the peroxidase substrate 3,3′,5,5′-Tetramethylbenzidine (Sigma-Aldrich, St. Louis, MO) was added to the plate to detect antigen bound biotinylated palivizumab. The remainder of the assay was continued as described above. Wells containing biotinylated palivizumab in buffer represented the un-competed positive control and wells containing PBS alone without any biotin labeled palivizumab were used as negative controls in the assay. Data was analyzed using SoftMax pro software (Molecular Devices, Exton, PA). Competitive binding titers were expressed as the 50% inhibition titers. Percent inhibition titers were calculated for each serum dilution using the following formula: (OD palivizumab - OD sample/OD palivizumab) × 100%. Using a 4-parameter fit curve analysis the titers were determined as the reciprocal value of the serum dilution that resulted in 50% inhibition of palivizumab binding. If 50% inhibition could not be obtained, a titer of <20 was recorded.

### Surface Plasmon Resonance

Biacore ™ T200 instrument (GE Healthcare, Baltimore, MD) was used to measure binding kinetics and affinity of palivizumab to purified RSV F nanoparticles. The surface of Sensor Chip CM5 used for capture was prepared using a human antibody capture kit (GE Healthcare). Blank sensor chip was prepared containing no antibody under identical coupling conditions for use as a reference. To capture palivizumab monoclonal antibody, 10 µg/ml was injected over the sample and references chips then gently washed. Duplicate samples of purified recombinant RSV F nanoparticles (100 nM) were injected over test and reference surfaces and binding curves generated after subtraction of the blank surface and blank injection (no analyte). The apparent equilibrium constant (K_d_) was calculated using the equation K_d_ = K_off_/K_on_.

### Serum Neutralization Assay

Serum was obtained from immunized cotton rats at day 49 and analyzed for neutralizing antibodies against RSV A Long virus in HEp-2 cells. Serial dilutions, starting at 1∶10, were prepared in 96 well plates. An equal volume (50 µl) of virus (200–300 pfu) was added to the diluted serum and the mixture incubated for 1 hr at 36°C. 100 µl of freshly trypsinized HEp-2 cells (5×10^5^ cells/ml) in growth medium (L-15, 10% fetal bovine serum and 2 mM glutamine) was added to the virus/serum mixture and the plates incubated for 6–7 days at 36°C or until positive control (virus only) wells show 100% cytopathic effect (CPE). Cells were scored for CPE microscopically, before and/or after, fixing and staining with 0.25% crystal violet in 5% glutaraldehyde. Stained plates were air-dried and evaluated for cytopathic effect using a dissecting microscope. The last dilution that resulted in 100% inhibition of CPE formation was identified as the endpoint neutralizing antibody titer for that sample. The assay limit of detection (LOD) = 10. Any sample resulting in a titer less than the LOD was assigned a value of 5. The geometric mean from each group was calculated ± SEM.

### Cotton Rat Protection

Five days after intranasal RSV challenge, cotton rats were sacrificed and the lungs harvested and weighed. Lung tissues were macerated and homogenized in 1 ml HMEM medium containing 10% SPG using disposable Kontes micro-homogenizer. The homogenate was clarified by centrifugation at 12,000×*g* for 10 min and virus titer in the supernatant was determined by plaque assay. Duplicate 250 µl volumes of the 10^−1^ and 10^−2^ dilution of the clarified homogenate was adsorbed for 2 hr on semi-confluent monolayers of HEp-2 cells in 24 well plates. Cells were overlaid with growth medium containing methycellulose (L-15, 5% fetal bovine serum, 2 mM glutamine and 0.75% methylcellulose) and incubated at 36°C for 6–7 days. At the end of the incubation period cells were fixed and stained with 0.25% crystal violet in 5% glutaraldehyde. Stained plates were air-dried and plaques counted after visualizing under a dissecting microscope. Results were expressed as plaques per gram of lung tissue. The arithmetic mean number of plaques for each group was calculated ± the SEM.

### Pulmonary Histopathology

Lung tissue was perfused with 10% formalin, embedded in paraffin and slides containing tissue sections (4 micron thickness) prepared. Tissue slides were stained with hematoxylin and eosin (H&E) and observed under a Nikon Eclipse microscope. Pictures were captured at 200X magnification using image Q capture and analyzed in Image Pro Plus software. Slides were evaluated in a blinded fashion using a score of 0 to 4 (0 = no; 1 = minimal; 2 = mild; 3 = moderate; 4 = maximum inflammation) in order of increasing severity for each of the following 5 parameters: a) peribronchiolitis; b) perivasculitis; c) bronchoiolitis; d) alveolitis and e) interstitial pneumonitis as described by Prince et al. [17]. The summary value for each of the five parameters was added together to arrive at a single summary score for each animal. Summary scores for animal in a group were used to arrive at the total average score/group expressed as the arithmetic mean±the SEM.

### Statistical Methods

Comparison between mean score of each group and non-immune animal challenge score was analyzed using Student’s t-test. The sum of the score of the five parameters per animal was used for analysis of histopathology data. The mean score of each group was compared to zero (non-immune animals challenge score) using ANOVA model with fixed effects for treatment group. Modeling was performed using PROC GLM in SAS.

## Results

### Modified Full Length RSV F

**Figure 1:**
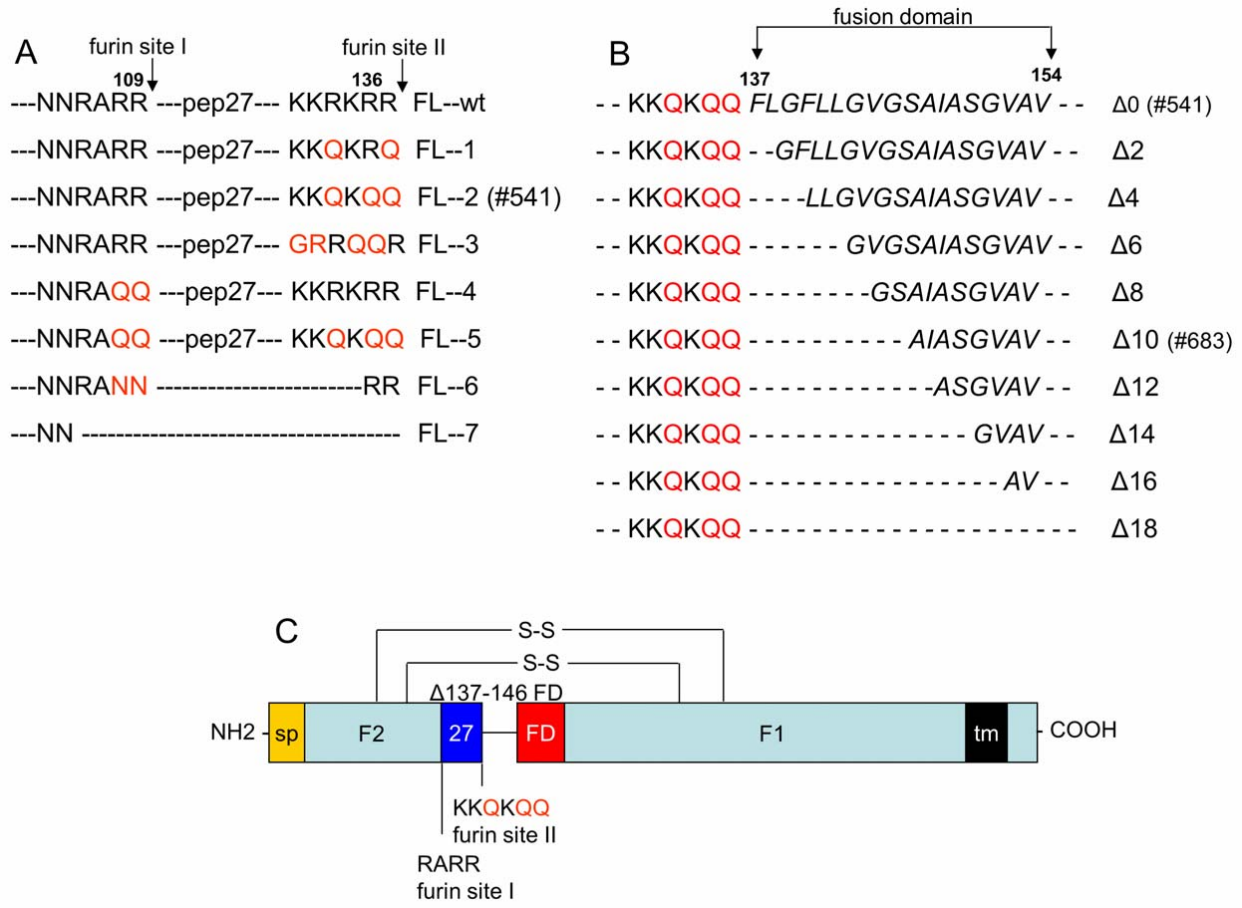
RSV A2 F gene modifications. (A) RSV F cleavage site mutation where introduced at the proteolytic furin cleavage site II (KKRKRR) and site I (RARR) of wild type RSV F gene (wt). (B) RSV F clone 2 (#514) with a mutation of cleavage site II was further modified with increasing deletions in the fusion peptide starting at Phe137 to Val154 with Δ2, Δ4, Δ6, Δ8, Δ10, Δ12, Δ14, Δ16 and Δ18 amino acid deletions. (C) The modified full length RSV F gene (#683) with a modified cleavage site II (KKQKQQ) and Phe137 - Ser146 fusion domain deletion including RSV F cleavable signal peptide (sp), 27 amino acid peptide [23], hydrophobic transmembrane (tm), and F2 and F1 polypeptides covalently linked by two disulfides.

Native, full length RSV F protein expression in the baculovirus insect cell expression system is very low, likely due to the toxicity from proteolytic cleavage and exposure of the F1 fusion domain (data not shown). To improve expression of RSV F in Sf9 cells a series of amino acid substitutions and deletions at or within furin cleavages site I at Arg109 and II at Arg136 and the F1 N-terminal hydrophobic fusion domain between Phe137 and Val154 were engineered (Figure 1). Multiple point mutations were introduced into cleavage sites site I (RARR) or II (KKRKRR) to generate five cleavage site mutation constructs (Figure 1A, clones 1 to 5). In addition, clones 6 and 7 were made by modifying or deleting both cleavage sites and the entire 27aa peptide sequence (pep27) between them (Figure 1A, clones 6 and 7). A total of seven cleavage site mutations were compared for expression of RSV F in Sf9 cells. Clones 1, 3, 4, 5, and 6 displayed either no improvement or decreased expression of F compared to wild-type RSV F. No RSV F was detected in Sf9 cells infected with a baculovirus expressing clone 7 where both furin cleavage sites and the intervening 27 amino acid peptide sequences were deleted (not shown). However, clone 2 (Figure A, #541) with furin site II modified from KKRKRR to KKQKQQ improved the expression of RSV F about 3-fold in Sf9 cells (data not shown).

**Figure 2:**
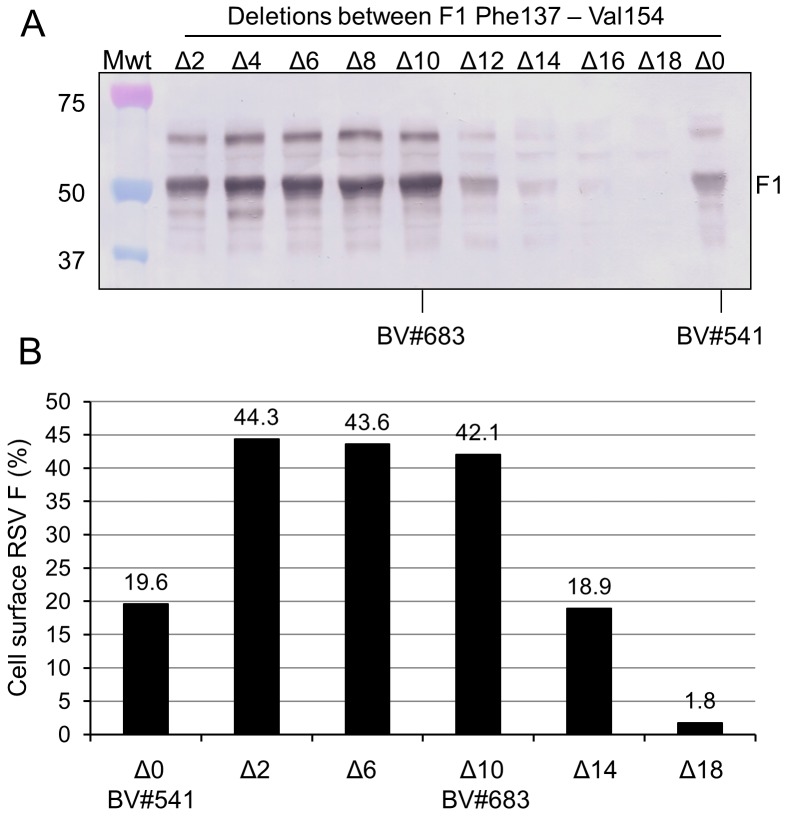
Expression of RSV F fusion domain mutants. Sf9 insect cells were infected at MOI = 1 with rBV expressing RSV F containing nine different fusion domain deletions (Δ2, Δ4, Δ6, Δ8, Δ10, Δ12, Δ14, Δ16, and Δ18) engineered into clone #541 with a modification of furin site II an intact fusion domain (Δ0). Infected cells harvested 67 hr post infection, extracted with buffer containing 0.5% Tergitol NP9 and soluble fractions analyzed by SDS-PAGE and Western blot. (A) Western blot analysis with anti-RSV F1 specific monoclonal antibody of nonionic detergent soluble proteins from Sf9 cells infected with baculovirus vectors containing RSV F genes with 2 to 18 amino acid deletions in the fusion domain from Phe137– Val154; each derived from RSV F #541 with a modification of furin site II. (B) Sf9 insect cells infected with rBV expressing the RSV F fusion domain deletions as described above were analyzed for cell surface RSV F protein immunostaining with anti-RSV F hyper-immune sheep serum, followed by staining with PE conjugated donkey anti-sheep IgG and FACS analysis.

To further increase the RSV F protein expression, a series of deletions in the F1 fusion domain of Δ2, Δ4, Δ6, Δ8, Δ10, Δ12, Δ14, Δ16 and Δ18 amino acids from Phe137 - Val154 were introduced into clone #541 (Figure 1B). Sf9 cells infected with baculovirus expressing these deletion mutants were analyzed for RSV F protein extracted from infected cells with a non-ionic detergent (Figure 2A) and by FACS analysis of cells stained for RSV F (Figure 2B). Deletions of up to 10 amino acids Phe137– Ser146 (Figure 2A Δ2, Δ4, Δ6, Δ8 and Δ10) increased the level of soluble F1 extracted from the cells relative to the parent clone BV#541 (Figure 2A, lane Δ0). A dramatic loss in soluble RSV F was observed with increasing deletions of the fusion domain greater than 10 amino acids likely due to miss-folding of the molecule (Figure 2A). Consistent with these results were constructs with Δ2, Δ6 and Δ10 amino acid deletions of the F1 fusion peptide displayed the highest cell surface expression of F (Figure 2B).

Baculovirus vector BV#683, containing a mutation in furin cleavage site II (KKQKQQ to KKRKRR) and a 10 amino acids deletion (Phe137 - Val146) in fusion domain (Figure 1C) was selected for further development.

### Purification RSV F

**Figure 3:**
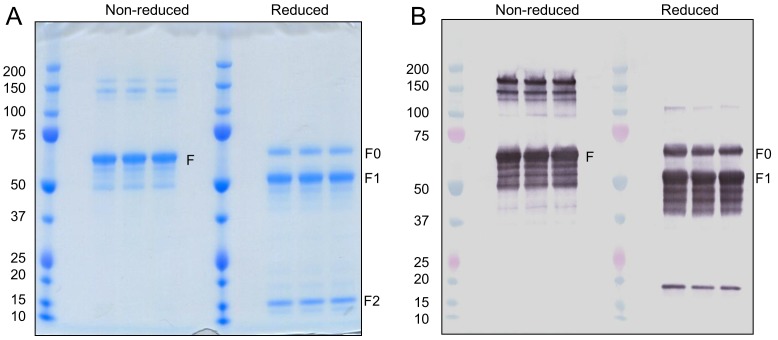
SDS-PAGE and Western blot analysis purified RSV F. Sf9 insect cells were infected at MOI = 1 with BV683 expressing modified full length RSV F and harvested 67 hr post infection and purified as described in the Methods. Purified RSV F protein was analyzed for purity by SDS-PAGE under non-reducing or reducing conditions to retain or disrupt intra-protein disulfide bond between F1 and F2 subunit. (A) Coomassie blue strained gel of replicate samples of non-reduced or reduced purified RSV F. (B) Western blot using anti-RSV F1 monoclonal antibody of non-reduced and reduced RSV F. The location of non-reduced polypeptide (F), reduced but full length (F0), and F1 and F2 polypeptides are indicated with protein marker molecular weight x 10^3^ included in the first lane.

Sf9 cells were infected with the baculovirus BV#683 and three days post infection RSV F was extracted from infected cells with a non-ionic detergent and purified using anion exchange, lectin affinity, and cation exchange chromatography. Purified F analyzed using SDS-PAGE and Western blot under non-reducing conditions was composed of a major 62 kDa polypeptide recognized by an anti-RSV F monoclonal antibody to F1 (Figure 3A and 3B; left panels). Immunoreactive bands just below F may represent different glycoforms or, as discussed below, a small fraction of F1 that was not covalently linked to F2 and the higher molecular weight polypeptides maybe oligomers of F not completely disrupted during non-reducing SDS-PAGE. Treatment of purified F with reducing conditions generated three major polypeptides; 62 kDa, 50 kDa and 12 kDa (Figure 3A; right panel). These were sizes predicted for uncleaved RSV F0 (62 kDa) and cleaved F1 (50 kDa) and F2 (12 kDa) polypeptides. F0 and F1 were reactive with the anti-RSV F1 monoclonal antibody (Figure 3B; right panel). A minor 18 kDa immunoreactive polypeptide was likely a proteolytic fragment of F1 (Figure 3B; right panel). About 5% of the antigen migrated as F0 under reducing conditions (Figure 3A and 3B; right panel) and was present even after reduction and alkylation (not shown).

### Electron Microscopy, HPLC, Dynamic Light Scattering, and Plasmon Resonance RSV F Nanoparticles

**Figure 4:**
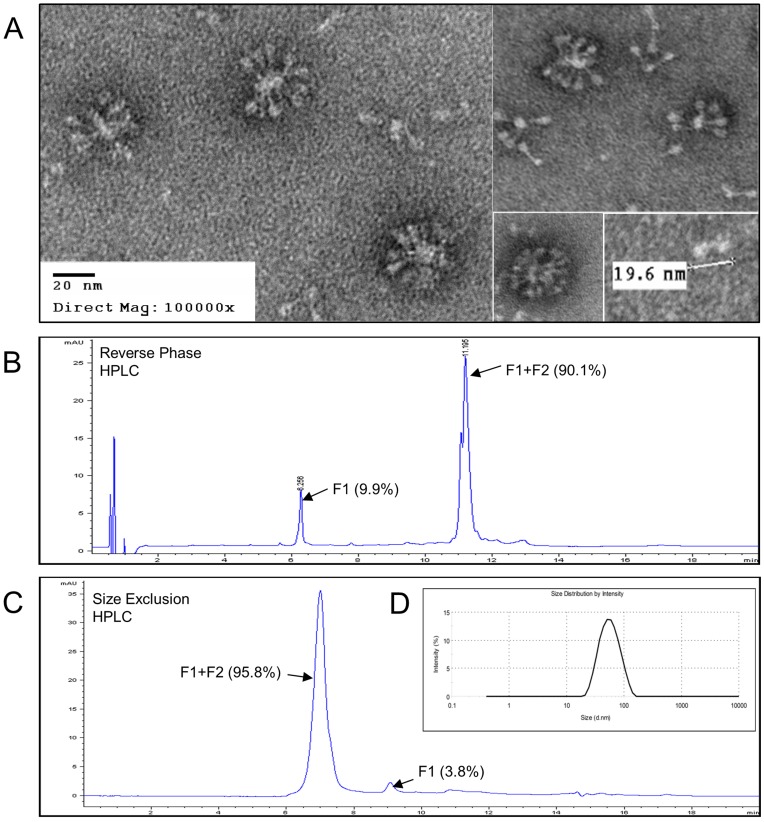
EM, HPLC, and DLS analysis of purified RSV F. (A) Negative staining electronmicroscopy of purified RSV F produced in Sf9 cells with the baculovirus vector BV683. RSV F nanoparticles were adsorbed to carbon parlodion-coated copper grids and negatively stained with 1% phosphotungstic acid. Bar represent 20 nm. (B) Reverse phase HPLC analysis on a PLRP-S 4000A, 2.1×150 mm column purified RSV F consisted of 90.1% F1+F2 at retention time of 11.195 min and 9.9% F1 at retention time of 6.256. (C) RSV vaccine particle size estimation by size exclusion HPLC RSV F protein using a BEH200, 1.7 µm, 4.6×300 mm size exclusion column. Size exclusion chromatography showed the RSV F nanoparticles consist primarily of F0 (covalently linked F1 and F2) with a low level of free F1 subunits. F0 (F1+F2) peak is 95.8% and F1 peak is 3.8% of the total peak area. (D) Dynamic light scattering of purified RSV F showing the distribution (%) of the intensity of light scattered by the particles using a detector placed at an angle 173°. Relative light intensity (%) was plotted relative to the particle diameter in nanometers (d.nm) of RSV F.

Negative staining electron microscopy of purified recombinant RSV F showed a distribution of about 40 nm diameter particles formed from multiple RSV F trimers (Figure 4A). The oligomers were hatpin-shaped rods with the wider end projecting away from the centers, consistent with what has been reported for the postfusion conformation of RSV F [12]–[14], [18]. A single F trimer was 19.6 nm in length with a triangular end view, characteristic of F trimers [18].

Reverse phase high pressure liquid chromatography (RP-HPLC) analysis show purified RSV F micelles consists of 90.1% F1+F2 at retention time of 11.195 min and 9.9% F1 at retention time of 6.256 (Figure 4B). The F1+F2 peak at 11.195 min showed a double peak, which may suggest different glycosylation species. The identity of F1+F2 and F1 were confirmed by SDS-PAGE and Western blot analysis of isolated HPLC peak fractions (data not shown). Intact mass determination by mass spectrometry showed F1 and F1+F2 having a molecular weight of 50 KDa and 61 KDa respectively, similar to the predicted molecular weight.

Purified RSV F nanoparticles were further analyzed using HPLC size exclusion chromatography (SEC). SEC analysis showed the RSV F nanoparticles consist primarily of covalently linked F1 and F2 (Figure 4C; F1+F2) with a low level of free F1 subunits with F1+F2 95.8% and F1 3.8% of the total peak area. The purity of RSV F particles was estimated to be ≥98%. F1+F2 peak was eluted in the void volume of this SEC column and F1 peak had a mass of about 180 KDa as expected for F1 trimers. Analytical ultracentrifugation (AUC) study showed the majority of species in RSV F nanoparticles have the molecular weight between 1–8 million Da (data not shown).

Particle size value for purified RSV F nanoparticles measured using dynamic light scattering was 32.3 nm with a pdi of 0.188 and width of 12.5 nm. A pdi value lower than 0.2 suggests the sample was relatively homogenous and consisted of a monodispersed species of particles and no evidence of significant aggregation (Figure 4D). Particle size was not measured for RSV F nanoparticles in the presence of Alum since the mixture forms large micron sized particles due to the nature of the Alum. Potency values and SDS-PAGE gels run on RSV F protein desorbed from RSV F Alum mixture (using a desorption method developed in-house) did however show a consistent potency value and gel band pattern as seen for RSV F nanoparticles without Alum (data not shown). Desorption of the RSV F protein was achieved by incubating the RSV F Alum mixture for one hour in a phosphate buffered solution containing 10% sodium citrate and 1.0 M sodium chloride.

The apparent binding affinity of the palivizumab to purified RSV F nanoparticles as measured using surface plasmon resonance was less than one nanomolar (K_d_ = 0.22 nM), consistent with what has been reported for postfusion RSV F [13].

### Immunogenicity and Protection in Cotton Rats

The immunogenicity of the RSV F micelle vaccine was evaluated in cotton rats. On day 0 and 21 animals were vaccinated i.m. with one of three doses of RSV F (1, 6, and 30 µg) in the absence or presence of aluminum phosphate adjuvant. Additional groups included animals immunized with formalin inactivated RSV (FI-RSV) and control animal vaccinated with PBS. The RSV positive control group received a single intranasal vaccination on day 0 with 1×10^6^ pfu of RSV Long strain. RSV F-specific antibody titers were evaluated in serum samples obtained 28 days following the second immunization. Immunization at all doses of unadjuvanted RSV F nanoparticles resulted in high titers of anti-RSV F serum antibody. Antibody titers obtained after two doses of 1.0 µg RSV F in the absence of adjuvant were found to be significantly higher (p<0.001, t-test) than those obtained following RSV i.n. infection. Co-administration of aluminum phosphate adjuvant with the RSV F nanoparticles enhanced vaccine immunogenicity and resulted in increases in geometric mean anti-RSF F antibody titers of 7–17 fold.

**Figure 5:**
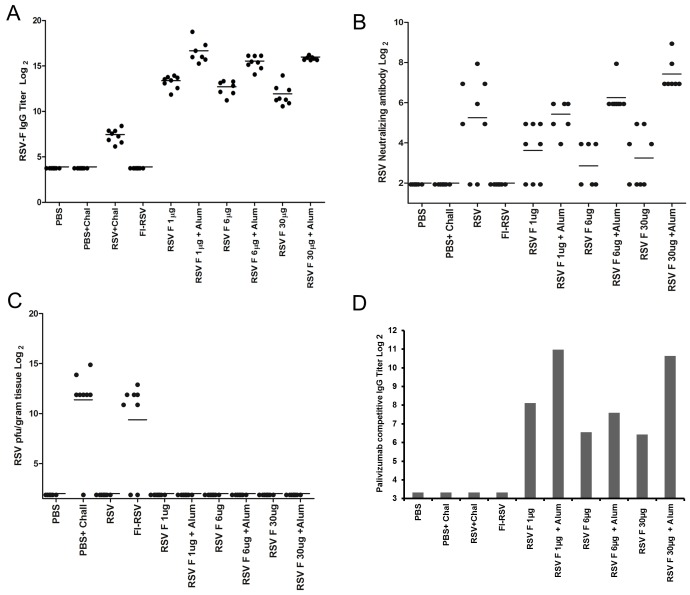
Cotton rat challenge model. Cotton rats (n = 8 per group) were immunized on day 0 and 21 with FI-RSV, PBS or the indicated doses of RSV F nanoparticle vaccine in the presence or absence of aluminum phosphate (alum). Negative control animals were immunized with PBS and not challenged (PBS). Positive controls were immunized with PBS then challenged with 1×10^6^ pfu RSV (PBS+Chal) or received a single intranasal challenge with 1×10^6^ pfu RSV on day 0 then challenged with RSV (RSV+Chal). Blood was drawn on day 49 from all animals for immunological analysis. (A) Day 21 serum anti-RSV F IgG ELISA titers against the purified recombinant RSV F nanoparticle. Titers are expressed as log2 and the geometric mean titer in each group marked with a bar. (B) RSV serum neutralizing antibody titers against RSV A2 providing 100% inhibition of CPE expressed as log2 and the geometric mean titer in each group marked with a bar. (C) Viral lung titers were determined 5 days post-challenge with RSV from homogenized lung tissue by plaque assay in HEp2 cells. Values represent RSV plaque forming units/gram lung tissue expressed as log2 pfu/g of lung tissue with the geometric mean titer in each group marked with a bar (D) palivizumab competitive IgG titers from pooled sera were determined as described in the Methods. Competitive titers (log2) were the reciprocal value of serum dilution that resulted in 50% inhibition of palivizumab monoclonal antibody binding to recombinant RSV F. In cases when the 50% inhibition could not be obtained, a titer of <20 was reported for the sample.

Cotton rats infected with live RSV mounted a significant but lower anti-RSV F IgG response in each of the infected animals (Figure 5A). Immunization with FI-RSV failed to induce detectable anti-RSV F antibody (Figure 5A) when measured against purified RSV F nanoparticles, however was positive when tested in ELISA against purified RSV virus (not shown).

Functional activity of anti-RSV F antibody was evaluated in a RSV neutralization assay in which the dilution of antisera capable of inhibiting 100% RSV cytopathic activity on HEp-2 cell monolayers was determined. Immunization with all doses of unadjuvanted RSV F nanoparticles resulted in the development of RSV neutralizing antibody (Figure 5B). Consistent with results for the anti-RSV F IgG, co-administration of aluminum phosphate adjuvant increased RSV neutralizing antibody titers 3.5–18 fold. Neutralizing antibody titers induced with live RSV were comparable to the low dose RSV F adjuvanted group and likely a component was directed against the RSV G protein. No neutralizing activity was observed with the cotton rats immunized with formalin inactivated RSV (Figure 5B; FI-RSV).

The *in vivo* effect of anti-RSV activity induced by immunization with RSV F nanoparticles was assessed by determining the level of viral replication in the lungs of cotton rats challenged intranasally with 1×10^6^ pfu RSV Long strain. Lung tissue from challenged animals was collected 5 days post challenge, homogenized and virus titers determined by plaque assay (Figure 5C). No virus could be detected in lung tissue obtained from cotton rats immunized either by i.n. delivery of live RSV or with any doses of RSV F nanoparticles administered either in the presence or absence of aluminum phosphate. When compared to the virus yield obtained from PBS immunized control and FI-RSV immunized animals (GMT = 9,375 and 3,125 pfu/gram respectively). These results indicate that neutralizing antibody elicited by RSV F nanoparticles is highly effective in preventing RSV virus replication in the lungs of challenged animals.

### Induction of Palivizumab Competing Antibody

Antigenic site II on the RSV F protein has been shown to be the target of palivizumab, a humanized RSV neutralizing monoclonal antibody used in prophylaxis for the prevention of RSV disease [19]. To determine whether antibodies directed against antigenic site II were induced by immunization with RSV F nanoparticles, a palivizumab competitive ELISA was performed. Sera from individual animals within each group were pooled and the ability to inhibit the binding of palivizumab monoclonal antibody to the purified RSV F nanoparticles measured. Inhibition of palivizumab binding was not observed with sera obtained from either live RSV, FI-RSV immunized animals, or PBS control animals (Figure 5D). All serum pools obtained from animals immunized with RSV F nanoparticles either in the presence or absence of aluminum phosphate had high levels of antibody that inhibit the binding of palivizumab to RSV F (Figure 5B).

### Histopathology Challenged Cotton Rats

The FI-RSV induced enhanced disease exacerbation (ERD) in infants is characterized as bronchoconstriction and severe pneumonia with peribronchiolar inflammatory cell infiltrates containing eosinophils, mononuclear cells and polymorphonuclear cells [1]. This consequence has been replicated in the cotton rat [17]which serves as a model for vaccine disease exacerbation. To determine whether immunization with the RSV F micelle vaccine resulted in disease exacerbation, lung histopathology was assessed following intranasal RSV challenge of immunized animals. Histological hematoxylin and eosin stained slides were prepared from lung tissue 5 days post RSV challenge and evaluated for peribronchiolitis, perivasculitis, bronchoiolitis, alveolitis and interstitial pneumonitis. While eosinophilia were reported in human ERD pathologic sections [1], recent review of this data indicates that eosinophils were infrequent and the predominant finding was neutrophilia [20]. Eosinophils are observed in mouse RSV ERD model [21] but were not reported in the cotton rat model [17] suggesting that the mouse model does not faithfully replicate the ERD pathology.

**Figure 6:**
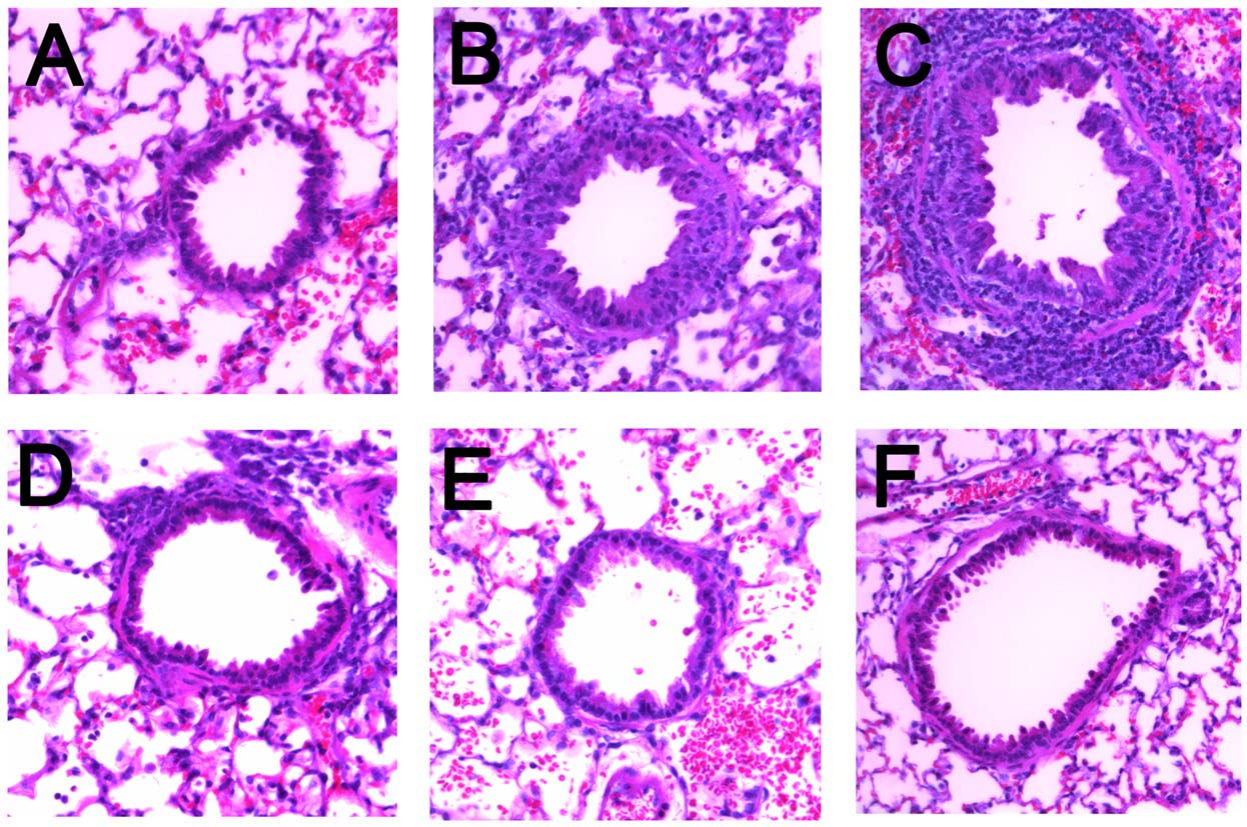
Cotton rat lung histopathology. Cotton rats were immunized on day 0 and 21 and challenged intranasally with 1×10^6^ pfu RSV A Long strain virus on day 49. Lung tissues harvested 5 days post challenge were fixed in 10% buffered formalin, paraffin embedded, sectioned and stained with hematoxylin and eosin. A: PBS vaccine-no RSV challenge; B: PBS vaccine-RSV challenge; C: FI-RSV vaccine - RSV challenge; D: 1 µg RSV-F vaccine with alum - RSV challenge; E: 6 µg RSV-F vaccine with alum - RSV challenge; F: 30 µg RSV-F vaccine with alum - RSV challenge.

Post-challenge lung sections from cotton rats immunized with 1, 6 or 30 µg RSV F nanoparticle vaccine±alum showed reduced pulmonary pathology compared to PBS and FI-RSV immunized animals. Representative H&E tissue sections of alveoli and bronchioles and surrounding tissue from vaccinated and control cotton rats are shown in Figure 6. After RSV challenge, the FI-RSV and PBS group had alveolitis, peribronchiolitis and lymphocytic infiltrates around the bronchioles, with the greatest level of alveolitis see in the FI-RSV group. A total histopathology score (sum of scores for the five individual evaluations described in the Materials and Methods) for each animal was determined and the mean of all measures of disease calculated. Analysis of the histopathology scores for the various experimental groups (Table 1) demonstrated FI-RSV exacerbated inflammation upon subsequent challenge with live virus. While FI-RSV histopathology shows the presence of occasional eosinophils, eosinophilia was not observed. The post challenge histopathology score for FI-RSV was significantly higher than that observed for non-immune animals challenged with RSV (mean 5.63; p<0.0001, t-test). No significant increase in pathology was observed in any RSV F immunized group either in the presence or absence of alum relative to the control groups. And, the lowest histopathology scores of 1.13 and 1.43 were in the adjuvanted vaccine groups immunized respectively with 6 µg or 30 µg RSV F nanoparticles and the RSV infected then challenged cotton rats which had a mean score of 0.75 (Table 1).

**Table 1:** Lung Histopathology Cotton Rats.

Total Score for Each Histological Parameter						
Treatment	Bronchiolitis	Vasculitis	Bronchitis	Alveolitis	Pneumonitis	Mean, all measures
PBS alone	0	0	1	0	0	0.13
PBS+Chal	5	0	10	5	1	2.63
RSV+Chal	1	1	2	2	0	0.75
FI-RSV+Chal	15	0	19	9	2	5.63
1 ug RSV-F	6	0	9	2	0	2.13
1 ug RSV-F+ alum	7	5	7	5	0	3.43
6 ug RSV-F	5	0	11	3	0	2.71
6 ug RSV-F+ alum	0	0	7	2	0	1.13
30 ug RSV-F	6	2	13	6	1	3.50
30 ug RSV-F+ alum	1	0	6	3	0	1.43

Lung histopathology scores from cotton rats immunized with the indicated dose of RSV F (RSV-F) nanoparticles without or with aluminum phosphate adjuvant (RSV-F+alum), live RSV then challenged (RSV+Chal), PBS then challenged (PBS+Chal), or PBS alone. Cotton rats (n = 7 or 8 per group) were immunized on day 0 and day 21 and challenged with RSV virus on day 49. Lung tissues were harvested on day 54 and subjected to histopathology and blinded samples scored for severity on a scale of 0 to 4. Total and mean of all measures per animal for bronchiolitis, perivasculitis, bronchitis, alveolitis and pneumonitis are given.

## Discussion

A baculovirus construct was engineered to contain RSV A2 F gene with a partial truncation at the N-terminus of the F1 hydrophobic fusion domain and modification of one of two proteolytic cleavage sites proximal to the N-terminus of F1. Recombinant RSF F was glycosylated, cleaved into disulfide-linked F2 and F1 subunits, and efficiently expressed on the surface of Sf9 cells as membrane associated homotrimers. A process was developed to solubilize Sf9 cell membranes with non-ionic detergent, separate RSV F-detergent complexes from cellular lipids and other host contaminants. Removal of the detergent resulted in recombinant RSV F oligomers forming protein-protein nanoparticles approximately 40 nm. When examined using electron microscopy, essentially all of the recombinant F oligomers formed a distinct pin-like structure similar to that reported for RSV F expressed in HEp-2 mammalian cells [14]. Native RSV F is known to be metastable and can rearrange into lower energy post fusion structures that are rod shaped and reported to aggregate due to exposure of the hydrophobic F1 fusion peptide [22]. Deletions of 9 or 10 amino acids of the N-terminus of the F1 fusion peptide have also been reported to increase stability of postfusion F trimers [12], [23]. Our data indicate that deletions of up to 10 amino acids of the RSV F fusion domain, starting from the N-terminal Phe137 can significantly improve the efficiency of recombinant F expression on the surface of Sf9 insect cells. This improvement may be due to the reduced fusion activity contributed by partial deletion in F fusion domain (data not shown) and an increase in the stability of the recombinant RSV F following exposure of the hydrophobic fusion domain upon cleavage of F2 and F1. Regardless, the modification introduced into the RSV F construct selected for vaccine development did not remove or modify the hydrophobic transmembrane and endodomain of RSV F at the carboxyl terminus. This second hydrophobic region would be critical in proper membrane interaction and the formation of quaternary structures between multiple RSV F oligomers like those mapped for the RSV neutralizing antibody motavizumab which binds to postfusion RSV F at contact residues on two protomers [11], [12].

The cotton rat model was chosen to evaluate the immunogenicity of the RSV F nanoparticle vaccine as they are susceptible to human RSV [24] and the induction of neutralizing antibody can inhibit replication of RSV in the respiratory track following RSV challenge [25]. In this report we have demonstrated that RSV F nanoparticles were highly immunogenic and elicited high levels of neutralizing antibody. Data obtained in the mAb recognition study and from the palivizumab competition assay indicate that antigenic epitopes that define critical neutralizing sites on the viral F glycoprotein are retained on the RSV F nanoparticle. The *in vivo* protective efficacy of the RSV F recombinant nanoparticle vaccine is demonstrated by the inability to detect any replicating virus in the lungs of immunized animals following an intranasal challenge with RSV. While co-administration of aluminum phosphate with the micelle vaccine resulted in an enhancement in neutralizing antibody titers, the use of an adjuvant was not required to achieve complete protection.

Enhanced respiratory disease following natural infection was observed in children who received the FI-RSV vaccine in the 1960s [1], [26], [27] has been a major concern in the development of new RSV vaccines. Cotton rats have been used as a model to reproduce RSV disease exacerbation [17], [28] and were used here to evaluate the potential toxicities associated with the RSV F nanoparticle vaccine. Following an RSV challenge of animals immunized with purified RSV nanoparticles, either in the presence or absence of aluminum phosphate, no evidence of enhanced lung pathology was observed. In contrast formalin inactivated RSV (FI-RSV) immunized animals induced no detectable serum IgG against purified recombinant RSV F, or neutralizing antibody, or IgG that would inhibit the binding of the monoclonal neutralizing antibody to RSV F. However, FI-RSV immunized animals exhibited a significant increase in lung pathology compared to controls. The nature of the anti-RSV immune response that has been attributed to the failure of the FI-RSV is still in question. Studies have suggested that the lack of protection is due to disruption of key epitopes during formalin inactivation [17], [29], [30]. Delgado et al. [31] have shown that anti-FI RSV antibody responses exhibit a lack of antibody maturation due to poor toll-like receptor stimulation and suggest that low antibody avidity contribute to disease severity through the deposition of immune complexes identified in affected tissues [32]. RSV F nanoparticle vaccine induced high levels of neutralizing antibody associated with *in vivo* protective immunity and showed no evidence of disease.

This report describes the development of a modified full length RSV F efficiently produced in the baculovirus-insect cell expression system and, when purified, formed F oligomers with structures reported for a postfusion, six-helix bundle conformation [12]–[14], [18]. Because the hydrophobic C-terminal transmembrane region was left intact, RSV F oligomers formed protein-protein nanoparticles unlike, for example, a soluble RSV F with the fusion, transmembrane, and cytoplasmic domains deleted [13]. It should be noted that while presenting the RSF fusion protein in the form of protein nanoparticles may have a favorable impact on immunogenicity, RSV F nanoparticle and soluble antigens have not been directly compared. Structural studies have suggested that palivizumab and motavizumab neutralizing epitopes may be partially buried in the virus and require local dissociation of prefusion F for surface exposure of this important epitope [11], [12]. Consistent with this hypothesis is that antigenic site II maybe ‘cryptic’ in natural infections due to anti-F immunity to immature forms of RSV F released from lysed infected cells (32). However, the crystal structure of postfusion RSV F did not reveal steric hindrance for binding of palivizumab or motavizumab and palivizumab was shown to bind to F with a high affinity. The RSV F nanoparticle vaccine formed either postfusion or an intermediate postactivation form of RSV F that induced neutralizing antibodies that compete for binding of palivizumab antibody to RSV F. In addition, tight subnanomolar binding of palivizumab to purified RSV F nanoparticles were confirmed by surface plasmon resonance, consistent with the conformation-dependent antigenic site II being structurally correctly. Proper folding may be critical for an RSV F vaccine to induce palivizumab and motavizumab-like neutralizing antibodies as these epitopes may be dependent on RSV F quaternary structures. Together these results justified continued development and evaluation of the RSV F nanoparticle vaccine candidate in a phase 1 safety and immunogenicity trial in young adults (Glenn, et al., submitted for review).
